# Evaluating the impact of onsite diabetes education teams in primary care on clinical outcomes

**DOI:** 10.1186/s12875-020-01111-2

**Published:** 2020-03-03

**Authors:** Enza Gucciardi, Changchang Xu, Michele Vitale, Wendy Lou, Stacey Horodezny, Linda Dorado, Souraya Sidani, Baiju R. Shah

**Affiliations:** 1 School of Nutrition, Ryerson University, Toronto, ON Canada; 2 Dalla Lana School of Public Health, University of Toronto, Toronto, Canada; 3 Lunenfeld-Tanenbaum Research Institute, Sinai Health System, Toronto, Canada; 4 Modellicity Inc, Toronto, Canada; 5 Postdoctoral Research Fellow, School of Nutrition, Ryerson University, Toronto, Canada; 6 Adult Diabetes Programs, Trillium Health Partners, Mississauga, Canada; 7 Ryerson University, Toronto, Canada; 8 Daphnee Cockwell School of Nursing, Ryerson University, Toronto, Canada; 9 Department of Medicine, University of Toronto, Toronto, Canada; 10 Division of Endocrinology, Sunnybrook Health Sciences Centre, Toronto, Canada

**Keywords:** Diabetes education teams (DETs), Primary care, Clinical outcomes

## Abstract

**Background:**

To evaluate the impact of integrating diabetes education teams in primary care on glycemic control, lipid, and blood-pressure management in type 2 diabetes patients.

**Methods:**

A historical cohort design was used to assess the integration of teams comprising nurse and dietitian educators in 11 Ontario primary-care sites, which delivered individualized self-management education. Of the 771 adult patients with A1C ≥ 7% recruited, 487 patients attended appointments with the diabetes teams, while the remaining 284 patients did not. The intervention’s primary goal was to increase the proportion of patients with A1C ≤7%. Secondary goals were to reduce mean A1C, low-density lipoprotein, total cholesterol-high density lipoprotein, and diastolic and systolic blood pressure, as recommended by clinical-practice guidelines.

**Results:**

After 12 months, a higher proportion of intervention-group patients reached the target for A1C, compared with the control group. Mean A1C levels fell significantly among all patients, but the mean reduction was larger for the intervention group than the control group. Although more intervention-group patients reached targets for all clinical outcomes, the between-group differences were not statistically significant, except for A1C.

**Conclusions:**

Nurse and dietitian diabetes-education teams can have a clinically meaningful impact on patients’ ability to meet recommended A1C targets. Given the study’s historical cohort design, results are generalizable and applicable to day-to-day primary-care practice. Longer follow-up studies are needed to investigate whether the positive outcomes of the intervention are sustainable.

## Background

In Canada, diabetes self-management education (DSME) and support services are underutilized with uptake of only 25–30% of Canadians living with type 2 diabetes [1]. Uptake is even lower among older adults (age 65–79), low-income earners, recent immigrants, and those living with mental-health or other physical conditions, despite these people having potentially greater diabetes-management needs and receiving greater benefits [2]. Barriers to accessing education and support services often include low awareness of diabetes education programs (DEPs), unsuitable appointment times, long waiting lists, and inconvenient locations [3]. Physician referrals to education programs are also low, ranging from 14% [4] to 52% [5] in Canada, possibly because of physicians’ limited access to and low awareness of such programs [6].

As a result, most Canadians with diabetes receive care solely from their primary care providers (PCPs) [7], who note challenges in providing optimal diabetes care and self-management support [1, 5, 8]. Furthermore, fewer than half of Canadians achieve and maintain the recommended clinical targets for diabetes management, covering glycemic control, blood pressure, and lipids [7, 9]. Similarly, international studies show that 30–70% of patients with type 2 diabetes in primary care settings are not at target [10].

Given the complexity of diabetes management, patients need to be supported in primary care by either an interprofessional healthcare team with specific training in diabetes or diabetes specialists. This recommendation is based on recent meta-analyses that demonstrate how the provision of team care, and other various disease management and quality-improvement strategies (e.g. promotion of self-management and patient education), improve glycemic control and reduce cardiovascular risk [11]. Therefore, the creation of one-stop services, where patients receive both medical care and individualized self-management education, can address many of the access and uptake barriers reported in the literature [12].

Because many of these patients do not receive the care they need [13], in Ontario and across Canada, DEPs have started to collaborate with local PCPs by having teams of diabetes educators (nurses and dietitians) deliver specialized diabetes services at primary care sites. This collaboration between primary and specialty care allows PCPs and their patients access to certified diabetes educators who offer self-management training and support aligned with clinical practice guidelines [14] and the Chronic Care Model, which stresses the importance of cooperation and interaction between providers to help patients manage chronic diseases, including diabetes [15]. In this model, diabetes educators are more accessible to patients and support PCPs in the care of their patients’ diabetes management.

Yet, although integration of diabetes education teams (DETs) within primary care is spreading, evaluations are lacking in Canada and scarce internationally [16–23]. Of these few evaluations, most demonstrated positive benefits for patients. However, these studies targeted select groups of patients, such as those with poor glycemic control or at high risk for complications [20], used small samples [20, 22]**,** and have often investigated glycemic control (A1C) as the main outcome [16, 19, 22], even though this measure accounts for only part of the added cardiovascular risk faced by patients**.**

To address the above-described gaps in the literature, we evaluated the integration of diabetes-education teams in primary care by measuring its impact on several clinical targets for the management of diabetes: A1C, diastolic blood pressure (DBP), systolic blood pressure (SBP), low-density lipoprotein cholesterol (LDL-C), and the ratio between total cholesterol (TC) and high-density lipoprotein cholesterol (HDL-C). Specifically, our primary research goal was to assess whether more patients accessing the diabetes education teams onsite (intervention) reach clinical targets than in the control group (patients not accessing the diabetes education teams onsite) 1 year since its introduction. While our secondary goal was to assess whether mean improvements in clinical targets are seen 1 year after the intervention, compared with the control group.

## Methods

### Setting

Educator teams (i.e., a nurse and a dietitian), from three diabetes-education programs were sent to 11 primary care sites. All sites were located in urban areas, across a Southern region of Ontario, Canada from November 2009 to August 2014. Eight of the 11 primary care sites were family health teams (multidisciplinary teams of providers, which are usually comprised of nurses, dietitians, and pharmacists, alongside a family physician), two were family-medicine group practices (3+ family physician working together), and one was a solo physician practice.

### Study design

Educator teams had already been integrated into primary care sites in many regions in Ontario before the intervention commenced, precluding meaningful application of a randomized controlled trial or a cluster randomized trial [24]. Historical cohort design was used: patients receiving care from the educator teams represented the intervention group; whereas, others who did not at the same site served as the control group. Historical cohort designs with repeated measures are considered able to maintain validity of conclusions regarding intervention effects in which the patients belonging to two cohorts (those who did and did not participate in the intervention) are selected using the same eligibility criteria [25].

### Participants

Patients were recruited into the study if they had type 2 diabetes, were 18 years or older and had A1C ≥ 7 Overall, 771 patients were recruited; 487 patients were enrolled in the intervention and seen by the educator teams; the control group consisted of the remaining 284 patients. All intervention-group members were recruited from across sites. Even though eligible for the intervention based on the study inclusion criteria, control-group members were not referred by their PCPs or refused to attend and were randomly selected from a list of patients not referred to the onsite educator teams. In total, 406 patients were initially eligible as controls. Efforts were made to have an equal number of control and intervention patients from each research site as to not oversample from one site to avoid bias of the differences between sites; however, many sites did not have as many controls as intervention patients. When there were more controls than intervention patients, an online randomizer (www.randomizer.org) was used to generate a list of controls for us to select from. Some sites had smaller pools of control patients than others and we decided not to oversample sites with larger pools of control patients as to not introduce bias. The control patients’ index date was based upon the start date of diabetes teams at each primary care site. All control patients must have had a visit during two time intervals (1 year prior to and after the introduction of diabetes teams onsite). The study was approved by the Research Ethics Board. The need for consent was waived by the ethics committee and patient data were de-identified prior to data analysis.

### Data collection

Data were extracted from patients electronic medical records, kept at the participating site. Baseline demographic and clinical information (i.e., age, sex, duration of diabetes, smoking status, and comorbidity), treatment modality (i.e., diet, oral agents, insulin, or insulin with oral agents), medical treatment, and duration of insulin therapy were collected from patients’ charts 1 year before and after the intervention started. A few comorbidity-related variables were considered in the analysis; in particular, nine specific comorbidity variables (i.e., cardiovascular disease, hypercholesterolemia, hypertension, hyperlipidemia, mental health, nephropathy, neuropathy, obesity and retinopathy), classified as “1″ if the subject had the specific condition, or “0″ otherwise. In addition, a general comorbidity variable was also considered, which was classified as “1″ if the subject had any type of the comorbidity conditions, or “0″ if the patient did not have any of the comorbidity. Data on clinical outcomes (i.e., A1C, LDL-C, TC-HDL ratio, DBP and SBP) were also collected 1 year before and after the intervention began, at multiple time points (approximately every 3 months and up to 4 times) in each one-year period. For our first research goal, we measured the proportion of patients who achieved the recommended target for A1C (≤7%), LDL-C (< 2.0 mmol/L), TC-HDL ratio (< 4), DBP level (<130HHmg) or SBP level (<80HHmg). We also assessed change over time within and between patients in the intervention and control groups. For the secondary goal, we assessed mean change for A1C, LDL-C, TC-HDL ratio, DBP level or SBP level, within and between patients in the two groups.

### The intervention

The intervention primarily targeted patients with type 2 diabetes, who were newly diagnosed, had poor glycemic control, diabetes complications, or needed insulin. They were referred to the educator teams by their PCPs. Patients who required intense and specialized treatment, for example those patients with type 1 diabetes, gestational diabetes, or those on multiple daily insulin regimes, were referred to the local diabetes-education program. The diabetes-educator teams provided patients with self-management education, coaching, timely treatment adjustment (access to remote glycemic-regimen optimization and monitoring via telephone and email), and system-navigation support. DET members also provided PCPs with recommendations based on Clinical Practice Guidelines for optimizing medications and improving diabetes care, and supported PCPs’ decision-making processes about diabetes management. The teams visited sites either weekly or monthly, depending on patient case load at each site. Team members saw patients independently or collaboratively (depending on space availability), for approximately half an hour to assess patients’ level of diabetes self-care, diabetes knowledge, and lifestyle habits. The assessment results informed which supports were provided. All patients were encouraged to attend classes, workshops, cooking demonstrations, and grocery-store tours at their local education programs. Teams also provided medication-optimization recommendations and decision support for diabetes management to PCPs.

The educator teams developed treatment priorities and care plan in consultation with patients. Then these plans were shared with PCPs, who reinforced them on subsequent visits. PCPs and educators collaboratively managed patient care, for example, case conferences, when major changes to patients’ treatment plans (e.g., insulin initiation, prescriptions for supplies, dose titration) were considered. However, at some sites PCPs and educator teams were not concurrently on site, and therefore PCPs and educator teams communicated through written notes. Half-hour follow-up visits with the educator teams were scheduled over a one-year period for all patients, during which care plans, patient goals and needs were reviewed, discussed, and revised as necessary. However, the number and frequency of follow-up visits varied and were based on patient needs and the educator teams’ clinical judgment; for example, if a patient requested additional visits and/or required insulin or dose adjustment, or when a patient’s A1C was outside the target range. This kind of tailoring is typical for complex multilevel interventions implemented in real-world primary care practices. Patients in the control group did not receive care from the DETs and were seen by their physicians as per usual care (patients in the control group could not self-refer themselves to the DET services, as they needed a physician referral to access these services).

### Statistical methods

Continuous variables were tested for normality of distribution. Standardized mean difference (SMD) was used to compare patient-characteristic variables across the intervention and control groups. Chi-square test or t-tests were used to evaluate the difference in the proportion of patients reaching the A1C target or the mean A1C levels between the two groups, before and after the intervention. Paired t-tests were used to compare the mean clinical-outcome measures for each group before and after the intervention.

Clinical outcomes (e.g., whether a patient reached the A1C target [“1” for yes and “0” for no]), and the actual clinical measurements of the outcomes were aggregated over time to produce pre-intervention (12 months before) post-intervention (12 months after) values. Generalized Estimating Equations (GEE) approach (i.e., GENMOD procedure with “repeated subject” statement), assuming dependence among data for the same patient, was used to assess the effect of group and period (i.e., comparison within groups before and after the intervention) on the five clinical outcomes. Based on the backward stepwise selection and clinical relevance of the variables, six characteristic factors, i.e. age, years since diagnosis, sex, treatment modality, smoking status, and delivery-care model (also referred as “Site and Grouping”), were selected among all the patients characteristic, including any type of comorbidity, (as in Table 1) to adjust for the main effects (i.e., group and period) on the clinical outcomes in GEE models. For clinical interpretation, effect sizes were calculated.

**Table 1:** Distribution of patient characteristics by group

	Control (*n* = 284)		Intervention (*n* = 487)		Standard mean difference
Continuous variables	Mean	SE or Quartiles	Mean	SE or Quartiles	
Age	61.71	0.80	58.00	0.61	0.275
Years since diagnosis^a^	9.90	(3.27,13.99)	5.58	(0.83,11.00)	0.256
Categorical variables	n	%	n	%	
Sex					0.066
Male	151	53.17	275	56.47	
Female	133	46.83	212	43.53	
Treatment modality					0.238
Diet	44	15.55	52	10.68	
Orals	179	63.25	323	66.32	
Orals + Insulin / Insulin only	60	21.20	112	23	
Smoker^b^					0.104
Currently yes	49	18.15	104	22.41	
Currently no	221	81.85	360	77.59	
Site and grouping					0.270
Family health team affiliated with a hospital	135	47.54	295	60.57	
Family health team other	108	38.03	146	29.98	
Family group practice or solo practice	41	14.43	46	9.45	
Any type of comorbidity (Yes)	268	94.4	438	89.9	0.198
Specific comorbidity condition					
Cardiovascular disease (Yes)	81	30.1	98	22.4	0.199
Hypercholesterolemia (Yes)	49	`8.4	83	18.9	0.088
Hypertension (Yes)	182	67.7	271	61.9	0.153
Hyperlipidemia (Yes)	105	39	156	35.6	0.114
MH (Yes)	68	25.3	127	29	0.119
Nephropathy (Yes)	21	7.8	41	9.4	0.102
Neuropathy (Yes)	22	8.2	35	8	0.087
Obesity^c^ (Yes)	114	42.5	238	54.3	0.238
Retinopathy (Yes)	17	6.3	31	7.1	0.091

^a^Years since diagnosis has missing values. It includes only measurements for 187 patients in the control group and 358 patients in the intervention group

^b^Smoker has missing values. It includes only measurements for 270 patients in the control group and 464 patients in the intervention group

^c^Obesity as a comorbidity has missing values, it includes only measurements for 268 patients in the control group and 438 patients in the intervention group

The group-effect size (i.e., pre-post intervention change) was determined using the adjusted proportion or adjusted mean difference per standard deviation within each group. The intervention-effect size or the combined-effect size (i.e., change in intervention group versus that in control group) was generated by combining the group-effect sizes, and tested for statistical significance in difference using Rubin’s method [26]. As a supplement, to confirm the effect of intervention changes, a sensitivity analysis was performed by excluding two covariates with missing values (years since diagnosis and smoking status). This analysis took into consideration sample-size shrinkage (*n* = 522) after controlling for all six covariates. Additionally, propensity scores were estimated (using patient characteristics) for the patients and applied as covariates in the GEE regression, to verify the findings of main effects from the models. All analyses were performed using SAS 9.4 (SAS Institute, Cary, NC).

## Results

### Exploratory analysis on patient characteristics and clinical outcomes

Patients from the intervention group had an average of 2.4 visits, with a range of 1~8 visits (80% of the 487 intervention group subjects had 3 visits or less).The characteristics of the study sample (771 patients, of whom 487 received the intervention and 284 did not) are described in Table 1. There was no difference between intervention and control groups in sex and smoking status, as well as most of the comorbidity conditions (SMD < 0.2). Although there was a slight imbalance in the distribution of age, years since diagnosis, treatment modality, obesity, and delivery care model between the groups, the difference was small (SMD < 0.3) [27].

**Fig. 1:**
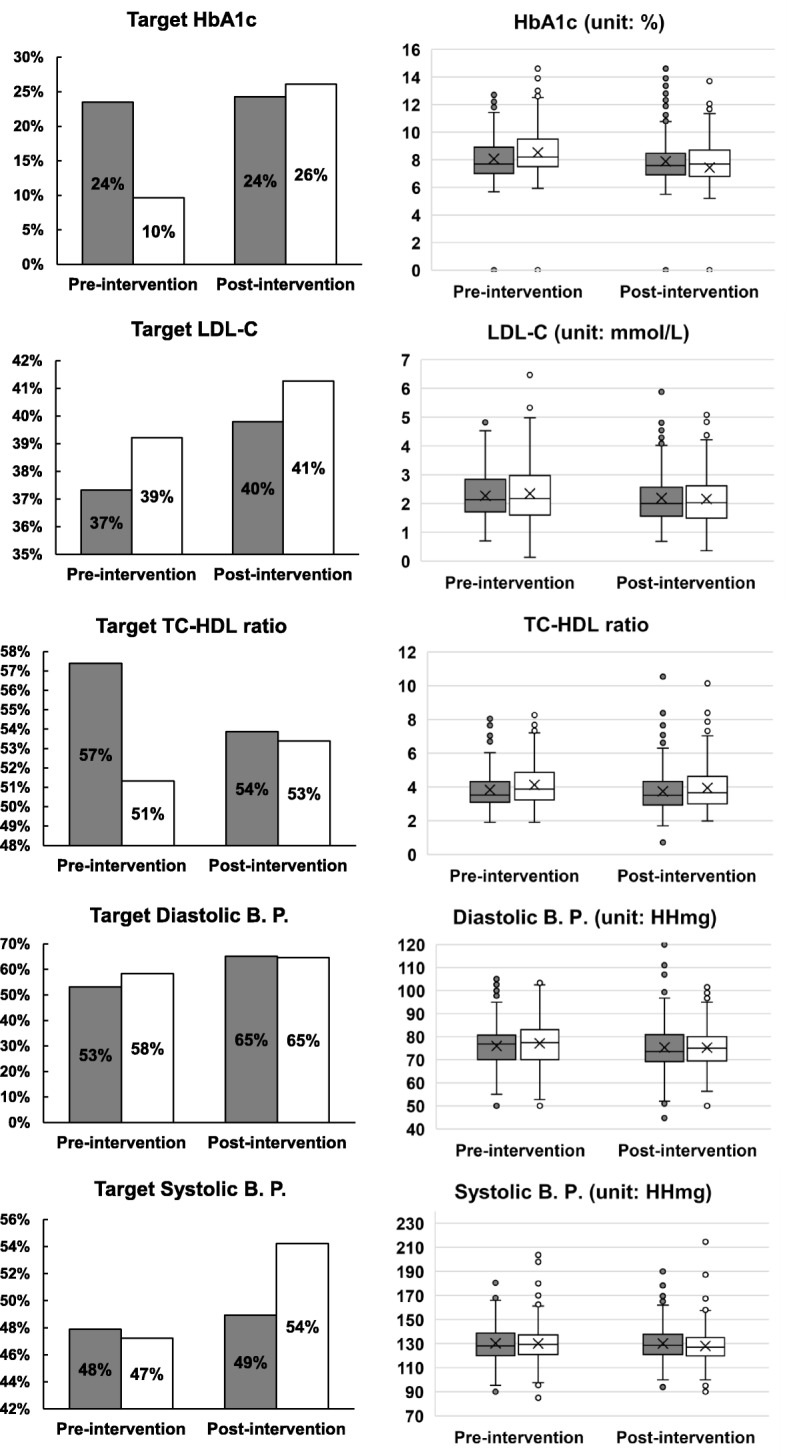
Percentage of patients reaching clinical targets before and after the intervention, by group (left-hand panels). Average clinical measurements before and after the intervention, by group (right-hand panels), in which X marks the mean and the solid line marks the median. Note: control group (*n* = 284) is in gray and the intervention group (*n* = 487) is in white

For both control and intervention groups, the by-period percentage of reaching target clinical outcomes and corresponding actual clinical measurements are illustrated in Fig. 1. The percentage of patients meeting the A1C target pre-intervention was significantly higher in the control group (*p* < 0.0001 by Chi-square test), suggesting an unbalanced distribution between the two groups pre-intervention. Post-intervention, this became non-significantly different, implying a greater increase in the proportion of patients at target for the intervention group. Comparing the individual means of clinical outcomes using paired t-test, highly significant improvement (*p* < 0.0001) was observed for all clinical outcomes in the intervention group; however, only some significant improvement for LDL-C (*p* = 0.043) and DBP (*p* = 0.048) was observed in the control group.

### Effect of the intervention on the change in primary-clinical outcomes

Using the GEE models, Table 2 compares the proportion of patients reaching targets within and between groups after adjusting for interaction between group and period, age, years since diagnosis of diabetes, sex, treatment, smoking status, site grouping, and clinical-outcome baseline value. The odds ratio (OR) within-group change is 3.849 with 95% CI [2.547, 5.816], indicating a more significant increase in the proportion of patients reaching A1C targets in the intervention group compared with the control group (OR = 1.150, 95% CI [0.772, 1.830]) through the intervention. This increase matches the findings in Fig. 1, before adjusting for any covariates, suggesting the intervention significantly helped patients reach the A1C target and lower their A1C levels.

**Table 2:** Adjusted differences in proportion of patients reaching clinical targets (expressed as odds ratios), within and between groups (GEE model, *n* = 522). Effect sizes for change in proportion of patients reaching targets within and between groups

	Covariates^a^ adjusted odds ratio (95% CI)				Effect size			
	Within group change (Post vs. Pre)		Between group difference by period (Intervention vs. Control)		Intervention group	Control group	Intervention vs. control	*p*-value
	Intervention	Control	Pre-intervention	Post-intervention				
A1C	3.849 (2.547, 5.816)	1.150 (0.722, 1.830)	0.330 (0.192, 0.567)	1.107 (0.710, 1.725)	1.279	1.031	1.24	0.02
LDL-C	1.099 (0.859, 1.405)	1.247 (0.875, 1.777)	1.180 (0.797, 1.746)	1.039 (0.702, 1.539)	1.029	1.067	0.965	0.697
TC-HDL ratio	1.115 (0.882, 1.411)	1.053 (0.710, 1.562)	1.020 (0.686, 1.516)	1.080 (0.717, 1.627)	1.036	1.014	1.021	0.817
DBP	1.182 (0.904, 1.545)	2.105 (1.443, 3.069)	1.544 (1.048, 2.273)	0.867 (0.568, 1.323)	1.033	1.147	0.901	0.074
SBP	1.464 (1.135, 1.888)	1.095 (0.788, 1.520)	0.887 (0.609, 1.290)	1.186 (0.822, 1.712)	1.119	1.029	1.088	0.359

^a^Adjusted for covariates (e.g., age, sex, years since diagnosis, treatment modality, smoking status and site-grouping)

Moreover, the combined effect size of the intervention versus the control group indicates that the increase in proportion of intervention-group patients reaching the A1C target is 1.24 times (*p* = 0.020) larger than the control group (Table 2). There appears to be a significant increase in the proportion of patients reaching the DBP target in the control group (OR = 2.105, 95% CI [1.443, 3.069]) than the intervention group, and a more significant increase in the proportion of patients reaching SBP in the intervention group (OR = 1.464, 95% CI [1.135, 1.888]) than the control group, but neither of their combined effect sizes was significant.

### Effect of intervention on the change in secondary-clinical outcomes

The changes in clinical mean values of the five outcomes were examined after adjusting for the same covariates as above, using GEE models. The intervention group exhibited significantly reduced average A1C (i.e., by − 0.007 with 95% CI [− 0.009, − 0.006]), but the control group did not have such change (95% CI not cover zero). The combined effect size of the intervention versus the control group also indicates that the mean reduction of A1C within the intervention group is significantly larger than the control group’s (*p* = 0.012) (Table 3). Although we did see more significant mean reduction of the other four clinical outcomes for the intervention group than the control group, none of their combined effect sizes are significant (Table 3).

**Table 3:** Adjusted differences in mean clinical-outcome measurements within and between groups (GEE model). Effect sizes for change of mean clinical-outcome measurements within the groups and within-group change across the two groups

	Within group change (Post vs. Pre)		Covariates^a^ adjusted odds ratio (95% CI)			Effect size		
			Between Group Difference by Period (Intervention vs. Control)		Intervention group	Control group	Intervention vs. Control	*p*-value
	Intervention	Control	Pre-intervention	Post-intervention				
A1C	−0.007 (−0.009, −0.006)	−0.002 (−0.004, 0.0001)	0.004 (0.002, 0.007)	− 0.001 (− 0.003, 0.001)	− 0.366	− 0.103	−0.262	0.012
LDL-C	−0.262 (− 0.339, − 0.184)	−0.082 (− 0.209, 0.044)	0.031 (− 0.121, 0.185)	−0.147 (− 0.304, 0.009)	−0.274	− 0.077	−0.197	0.083
TC-HDL ratio	−0.189 (− 0.288, − 0.090)	−0.073 (− 0.250, 0.103)	0.122 (− 0.135, 0.380)	0.006 (− 0.205, 0.218)	−0.15	− 0.038	−0.112	0.182
DBP	−2.048 (−2.972, −1.125)	−1.062 (−2.313, 0.189)	− 0.313 (−1.872, 1.246)	−1.300 (− 2.981, 0.380)	−0.171	− 0.091	−0.079	0.403
SBP	−2.226 (−3.550, −0.903)	− 0.990 (− 2.844, 0.862)	−0.653 (−3.293, 1.985)	−1.889 (−4.479, 0.699)	−0.131	− 0.058	−0.073	0.6

^a^Adjusted for covariates (e.g., age, sex, years since diagnosis, treatment modality, smoking status and site-grouping)

### Supplementary analyses

The sensitivity analyses were performed using the same GEE models as in Tables 2 and 3 and excluding the two covariates with missing values (years since diagnosis and smoking status), which showed findings very similar to the main effects (e.g., within-group change, and between-group difference) in the full models (i.e., Tables 2 and 3). The supplementary propensity score analysis, to assess the effect of the intervention on the clinical outcomes when free of potential confounding variables and biases, also produced results for all five outcomes that were highly consistent with the GEE models presented above. It is noted that when the comorbidity variables were included into propensity scores as covariates in the analysis, none of them significantly modified the effect of the intervention on the clinical outcomes as shown in the final GEE models. These results further confirm that the integration of diabetes education teams in primary care effectively lowers A1C levels.

## Discussion

Evidence shows that individuals with type 2 diabetes are not reaching clinical-outcome targets [7, 9, 10]. Yet, the integration of diabetes-education teams within primary care has the potential to increase patients’ access to and uptake of diabetes self-management training and counseling. This evaluation demonstrated that integrating diabetes-education teams into primary care sites significantly increased the proportion of patients reaching A1C targets. Although we found greater effect size for the intervention group on all clinical outcomes compared to the control group, it was only significantly greater for A1C. This is not surprising, as glycemic control is fundamental to the management of diabetes. Accordingly, physicians and diabetes teams are likely to treat hyperglycemia more aggressively than other cardiovascular risk factors, as lowering blood glucose is associated with fewer hospitalization for short-term complications [28] and potential short-term economic benefits [29].

### Primary-care-integrated education teams as a promising strategy

Several other studies have evaluated the impact of primary-care-integrated education teams, comprising nurses and dietitians on patients with type 2 diabetes. For instance, three randomized controlled trials found significant improvements in A1C levels for the intervention group at 6 months [22]; A1C, TC, and LDL-C levels at 6 months [30]; and for A1c and systolic blood pressure at 12 months [31]. A quasi-experimental study found significantly greater improvement in A1C levels in the intervention group after 12 months and no significant change for LDL cholesterol, and systolic and diastolic blood-pressure levels [18]. An observational study found improvements only in A1C and HDL-C levels, while LDL-C worsened, and blood pressure did not change significantly after 12 months [32]. Finally, pre-post design studies found that primary-care-integrated education teams improved glycemic control at 3 months [16], and at 12 months [17]. Overall, these evaluations generally reported positive results, and concur with our finding that primary-care-integrated education teams improve clinical outcomes among patients with type 2 diabetes, particularly glycemic control.

Following the indications provided by the Chronic Care Model, which emphasizes collaboration among health professionals to support and meet patients’ management goals [15], integration of specialized teams in primary care is growing, and can improve the quality of patient care [12]. For example, diabetes education teams can communicate patients’ needs for medication and dietary changes to their PCPs. Emphasizing shared rather than competing responsibilities, diabetes educators can identify knowledge and practice gaps at primary care sites, leading to better clinical assessment and implementation of diabetes-management guidelines [33]. Indeed, expanding educators’ role to include medication management, support and monitoring of individuals with diabetes is associated with improvements in glycemic control, and lower cholesterol levels and blood-pressure [34].

However, although integrating diabetes educators into primary care addresses fragmentation of care, it also comes with some challenges. First, primary care settings are only temporary homes for the visiting specialists. Space, resources, appropriate scheduling, and access to medical records must be negotiated within the contextual complexities of each setting’s functions and staff interactions [33]. Second, misuse and underuse of diabetes educators are also possible, especially in the initial stages of team integration, because diabetes educators’ scope and practice are unclear. To facilitate collaboration, it is important to institute formal orientation procedures, such as meetings that clarify the diabetes team’s services, roles, and responsibilities, and promote informal interactions between diabetes educators and family physicians to build reciprocal trust through face-to-face consultations [33].

### Limitations

Clinical outcomes other than glycemic control did not improve as expected. Because of the historical cohort design, testing for clinical-target outcomes were neither as frequent nor consistent across patients as it would be in a randomized controlled trial. For instance, fasting lipids (LDL-C, HDL-C, and TC) would be tested at the time of diagnosis, and, if results were initially normal, the tests would be repeated annually or as clinically indicated for each patient. If treatment for dyslipidemia begins, more frequent testing is warranted. Thus, inconsistent testing may have interfered with an accurate assessment of one-year change in our intervention and control groups. On the other hand, blood-pressure and A1C testing is recommended at every visit or at three to 6 months, so perhaps we observed more change in these variables because of the more frequent testing. More frequent testing (or a longer follow-up) is needed in order to observe change in a 1 year period.

Patients were also grouped based upon the natural referral patterns of the primary care physicians and thus, at baseline, intervention-group patients began with higher A1C levels and had lived with diabetes for less time than control-group patients, which represents a possible source of bias, as the literature shows that high-risk patients or those with comorbidities are more likely to be referred to diabetes educators [35]. However, we addressed the issue through further analysis: specifically, the propensity score incorporated regression. In addition, given the historical cohort design and access only to limited data collected from EMRs, we did not have data on patients’ self-care behaviours and lifestyles, which would have provided a better understanding of the proximal impacts of the exposure to diabetes educators. To better evaluate the impacts of the services provided collaboratively by physicians and diabetes educators, future studies should incorporate data on patients’ behaviours and lifestyles and adopt longer follow-ups to adequately assess change overtime. Despite these limitations, our study was a pragmatic trial where participant recruitment reflects variations among patients in real clinical settings to ensure generalizability and enhance external validity [36]. Although randomized controlled trials better control potential biases, their rigid design may reduce the generalizability of their results [37]. In contrast, observational studies can produce results as reliable as those of RCTs with appropriate sample size and follow-up [38].

## Conclusions

In summary, our evaluation demonstrated that integrating diabetes-education teams, comprising nurses and dietitians, into primary care settings can meaningfully impact patients’ ability to meet recommended A1C targets over a one-year period. It is common to evaluate interventions after 1 year [21, 32]; however, we suggest that longer-term evaluations, with more frequent measurements of cardiovascular risk factors, could better assess change of clinical outcomes over time and their long-term sustainability. Based on the positive outcomes demonstrated in our evaluation, this model is likely to succeed in other settings with similar medical-care resources, as a strategy to improve glycemic control among and support to patients living with type 2 diabetes.

## Data Availability

The datasets used and/or analyzed during the current study are available from the corresponding author on reasonable request.

## Abbreviations

DBP: Diastolic Blood Pressure
DEPs: Diabetes Education Programs
DETs: Diabetes Education Teams
DSME: Diabetes Self-Management Education
GEE: Generalized Estimating Equations
HDL-C: High-density Lipoprotein Cholesterol
LDL-C: Low-density Lipoprotein Cholesterol
PCPs: Primary Care Physicians
SBP: Systolic Blood Pressure
SMD: Standardized Mean Difference
TC: Total Cholesterol
